# Portable and Error-Free DNA-Based Data Storage

**DOI:** 10.1038/s41598-017-05188-1

**Published:** 2017-07-10

**Authors:** S. M. Hossein Tabatabaei Yazdi, Ryan Gabrys, Olgica Milenkovic

**Affiliations:** University of Illinois, Department of Electrical and Computer Engineering, Urbana, 61801 United States

**Keywords:** Nanobiotechnology, Information technology

## Abstract

DNA-based data storage is an emerging nonvolatile memory technology of potentially unprecedented density, durability, and replication efficiency. The basic system implementation steps include synthesizing DNA strings that contain user information and subsequently retrieving them via high-throughput sequencing technologies. Existing architectures enable reading and writing but do not offer random-access and error-free data recovery from low-cost, portable devices, which is crucial for making the storage technology competitive with classical recorders. Here we show for the first time that a portable, random-access platform may be implemented in practice using nanopore sequencers. The novelty of our approach is to design an integrated processing pipeline that encodes data to avoid costly synthesis and sequencing errors, enables random access through addressing, and leverages efficient portable sequencing via new iterative alignment and deletion error-correcting codes. Our work represents the only known random access DNA-based data storage system that uses error-prone nanopore sequencers, while still producing error-free readouts with the highest reported information rate. As such, it represents a crucial step towards practical employment of DNA molecules as storage media.

## Introduction

Modern data storage systems primarily rely on optical and magnetic media to record massive volumes of data that may be efficiently accessed, retrieved, and copied^1^. Key features of existing recorders include random access and highly accurate data retrieval, supported by low-cost, real-time operations. Recently, these systems were challenged by the emergence of the first DNA- and polymer-based data storage platforms^2–8^. These new platforms have the potential to overcome existing bottlenecks of classical recorders as they offer ultrahigh storage densities on the order of 10^15^–10^20^ bytes per gram of DNA^2–4, 6^.

Experiments have shown that using DNA-based data storage one can record files as large as 200 MB^6^, and ensure long-term data integrity through encapsulation^5^ and coding^4, 6, 9, 10^. Data retrieval has exclusively been performed via high-throughput, high-cost sequencers, such as Illumina HiSeq^2, 3^ and MiSeq^5, 6^, because inexpensive portable sequencers such as MinION may introduce a prohibitively large number of deletion, insertion, and substitution errors (Some highly conservative estimates^11^ for first-generation MinION sequencers suggested error rates as high as 30%, which by far exceed those of optical recorders equal to 1 bit/10 TBs^12^).

In order to make DNA-based data storage competitive with existing flash technologies, it is hence imperative to reduce *synthesis cost* by avoiding undesirable DNA sequence patterns; provide for *random access*, as otherwise selective reading becomes impossible; reduce sequencing cost by enabling *portable readout systems*; and offer *extremely low error rates*, comparable to those of classical recorders.

Our implementation addresses these challenges by introducing several unique, new concepts in bioinformatics, coding theory, and synthetic biology. In particular, it entails:

- Reducing the cost of synthesizing DNA containing user information via compression and subsequent constrained coding. Constrained coding eliminates substrings that may cause problems during synthesis, such as short repetitive substrings near the 3′ and 5′ ends of the string, it limits the length of homopolymers (homopolymers are “runs” of consecutive symbols of the same kind, for example, AAAA) that cause both synthesis and sequencing problems, and balances out the GC content within short substrings of the encoded data.
- Providing random access by storing data in gBlock codewords (long DNA strings) equipped with addresses that allow for accurate selection via polymerase chain reactions (PCRs). The addresses have specialized properties, such as GC balanced content, large mutual Hamming distance, and weak mutual correlation. Controlled mutual correlation allows for avoiding matches of substrings of the address sequences in encoded data, and consequent erroneous codeword selection. The addresses are constructed mathematically using two binary component codes, without resorting to computer search.
- Portability of the system is ensured by using nanopore sequencers, such as MinION, while error-tolerance, which is challenging to accomplish with such architectures, is built-in via a new set of consensus sequence construction algorithms and asymmetric deletion-correcting codes tailor-made for the nanopore channel. The new consensus method combines classical multiple sequence alignment methods with side information provided by the address sequences, and improves upon the state-of-the-art nanopolish platform, as it exploits the algebraic structure of the gBlock codewords. Furthermore, the deletion correcting codes are designed for errors that occur in consensus sequences, such as bounded magnitude errors in the homopolymer length sequences.

All these techniques are seamlessly combined into an integrated pipeline for *data encoding* (compression and constrained encoding) and *post-processing* (address sequence identification, iterative sequence alignment and error correction). On a broader scale, our work also presents experimental results regarding a new DNA-based data storage architecture that has many features of modern storage devices and paves the way for practical employment of macromolecular storage systems (See Table 1).

**Table 1:** Comparison of features/properties of current DNA-based storage platforms.

Work	Random access	Portability	Sequencing technology	Sequencer error rate	Error correction/detection	Net density (bits/bp)
Church^2^	No	No	HiSeq	0.1–0.3%	None	0.83
Goldman^3^	No	No	HiSeq	0.1%	Detection	0.33
Yazdi^4^	**Yes**	No	Sanger	0.05%	Correction	1.575
Grass^5^	No	No	MiSeq	0.1%	Correction	1.14
Bornholt^6^	Yes	No	MiSeq	0.1%	None	0.88
Erlich^9^	No	No	MiSeq	0.1%	None	1.55
This work	**Yes**	**Yes**	**MinION**	**12%**	Correction	**1.72**

## The Encoding Step

**Figure 1:**
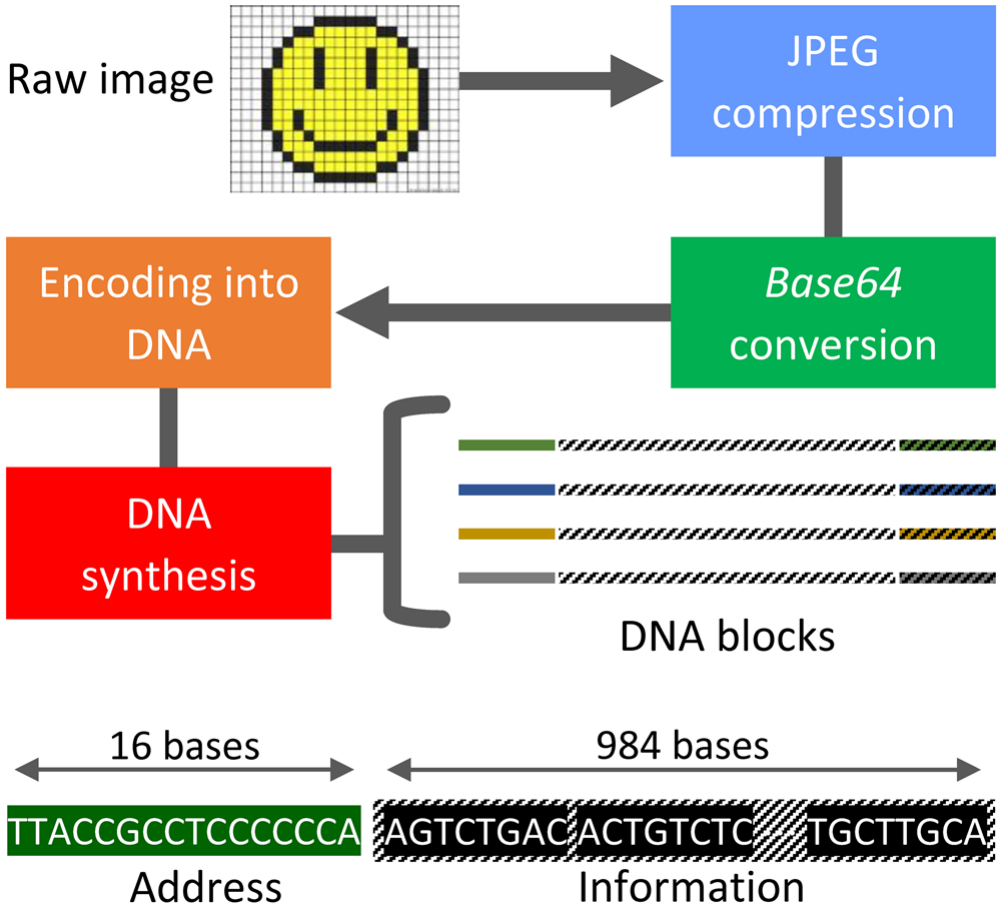
The encoding stage. This stage involves compression, representation conversion, encoding into DNA, and subsequent synthesis. Each synthesized DNA codeword is equipped with one or two addresses. The encoding phase entails constrained coding, which limits the occurrence of the address block to one predefined position in the codeword only, and GC-content balancing of each substring of eight bases. Additional homopolymer checks are added directly into the string or stored on classical media; they correspond to only 0.02% of the data content.

When compressed, data is stripped of its redundancy and errors in the compressed domain introduced either during synthesis or sequencing may cause catastrophic error propagation in the decompressed file. Even one single substitution error in the compressed domain may render the file unrecognizable. Hence, it may appear undesirable to perform data compression. Unfortunately, uncompressed files are significantly larger than their compressed counterparts, which implies significantly higher costs for synthesizing the information into DNA codewords. Our analysis detailed in the Supplementary Information shows, the cost of adding redundancy for eliminating errors in the compressive domain is negligible compared to the cost of synthesizing uncompressed files. As a result, to accommodate large file sizes at low synthesis cost, the data is first compressed. To introduce the redundancy needed for different stages of error correction and to minimize the addressing overhead, we chose the DNA codeword length to be 1,000 base pairs (bp). This codeword length also offers good assembly quality of long files without additional coverage redundancy or word identifiers, and the overall smallest commercial synthesis cost (the prevalent method for encoding information into DNA relies on the use of oligos of length close to 100 nucleotides. Such a length introduces high loss in coding efficiency when addressing is performed, and underutilizes nanopore sequencing platforms. Some work has reported lower synthesis cost for oligo sequences, but this may be due to special arrangements made with the companies performing synthesis). To accommodate this choice of codeword length, as well as the inclusion of codeword address sequences, we grouped 123 × 14 = 1,722 consecutive bits in the compressed file and translated them into DNA blocks comprising 123 × 8 = 984 bases. We then balanced the GC-content of each substring of 8 bases via specialized constrained coding techniques that extend our previous results in terms of mitigating the need for computer search and providing mathematical characterizations of the addresses^13^, outlined in the Supplementary Information. Balancing eliminates certain secondary structures, reduces synthesis errors, and helps to correct sequencing deletion errors. Each of the remaining 16 bases in a DNA codeword are used as a codeword address. As already pointed out, the purpose of the addressing method is to enable random access to codewords via highly selective PCR reactions. Selectivity is achieved by prohibiting the appearance of the address sequence anywhere in the encoded DNA blocks^4, 13^. Additional protection against deletion errors is provided via a new coding method we term *homopolymer check codes*. When coupled with balancing and subsequent read alignment steps, homopolymer checks lead to error-free readouts. A detailed description of the balancing and addressing schemes may be found in the Supplementary Information. Homopolymer checks are also discussed in the post-processing step. All the encoding techniques are universal and therefore transparent to the type of data to be stored. The encoding pipeline is illustrated in Fig. 1.

## The Post-processing Step

Post-processing follows the physical process of sequencing via nanopores, as outlined in the Supplementary Information. The reads obtained using the MinION MkI sequencers have sequence-dependent substitution, deletion, and insertion errors, described in detail in the Implementation Section. In practice, arbitrary combinations of deletions, insertions and substitution are harder to correct than deletions alone. Hence, we performed a consensus alignment procedure that “transforms” almost all insertion and substitution errors into deletion errors confined to homopolymers of certain lengths, and generates an estimate of the DNA codeword based on the noisy reads.

In the first phase of post processing, we constructed a rough estimate of the DNA codewords. For this purpose, we used the address sequences to identify high-quality reads, i.e., those reads that contain an exact match with the given address. Aligning all reads instead of only high quality reads results in a large number of errors, and the quality of the reads is highly nonuniform. Next, we ran different multiple sequence alignment (MSA) algorithms on the identified high-quality reads and obtained different consensus sequences. For that purpose, we used Kalign, Clustal Omega, Coffee, and MUSCLE^14, 15^. As multiple sequence alignment algorithms are traditionally designed for phylogenetic analysis, their parameters are inappropriate for modeling “mutations” introduced by nanopore sequencers. Hence, for each alignment method, new parameters were chosen by trial and error (see the Supplementary Information). The choice of the parameters was governed by the edit distance between the MSA consensus sequence and the corresponding DNA codeword.

As each alignment method produced a different consensus sequence, we formed an aggregate consensus. The aggregate consensus contains the “majority homopolymer” of the different MSA algorithms. As an example, if three MSA algorithms produced three consensus sequences, AAATTGCC, AATTTGCA, and AAATTGC, the majority homopolymer consensus would equal AAATTGCA, as two sequences contain a homopolymer of three As at the first position; two sequences contain a homopolymer of two Ts in the positions to follow; and all three sequences contain G and C. Observe that A is included in the last position of the consensus.

**Figure 2:**
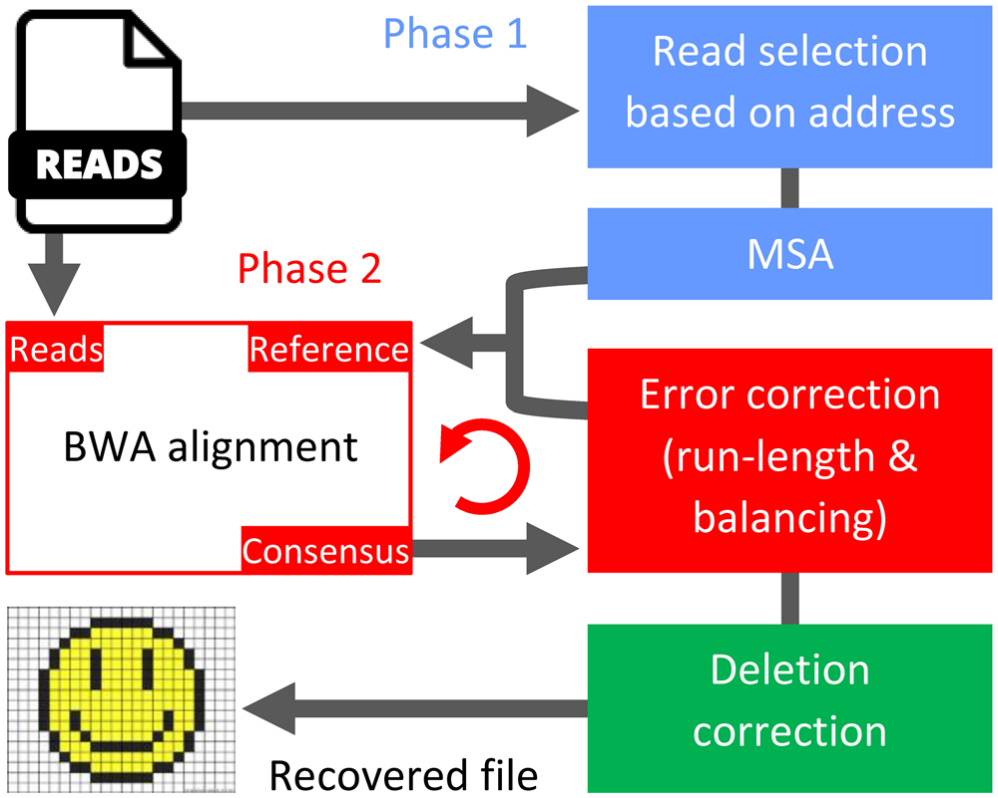
Post processing via sequence alignment and homopolymer correction. In the first phase, estimates of the DNA codewords are obtained by running several MSA algorithms on high-quality reads that contain an exact match with the address sequence. The second phase improves the estimate by employing an iterative method that includes BWA alignment and an errorcorrecting scheme.

In the second phase of post processing, we performed iterative alignment. By this stage, consensus sequences that estimate the original DNA blocks were identified, with errors mostly confined to deletions in homopolymers of length at least two. (See the Supplementary Information for a detailed analysis). To further improve the reconstruction quality of the blocks and thereby correct more errors, we performed one more round of BWA^16^ alignment to match more reads with the corresponding estimates of their DNA codewords. Once this alignment was generated, two sequential checks were performed simultaneously on the bases. The checks included computing the majority consensus for each homopolymer length and determining whether the GC-balancing constraint for all substrings of length 8 was satisfied. More precisely, in the majority count, only homopolymer lengths that resulted in a correct balance were considered. This procedure is illustrated by an example in the Supplementary Information. Note that alignment does not require any coding redundancy, while balancing uses *typical sequences* and, as a result of this, has a high coding rate of 0.88. The alignment procedure is depicted in Fig. 2.

In the final stage of post processing, we corrected deletion errors in homopolymers of length exceeding one. For this purpose, we used an error-correction scheme that parses the consensus sequence into homopolymers. As an example, the parsing of the sequence AATCCCGA into homopolymers AA, T, CCC, G, A gives rise to a homopolymer length sequence of 2,1,3,1,1. Special redundancy that protects against asymmetric substitution errors is incorporated into the homopolymer length sequence. If two deletions were to occur in the example consensus, resulting in ATCCGA, the homopolymer lengths would equal 1,1,2,1,1. Here, we can recover the original length sequence 2,1,3,1,1 from 1,1,2,1,1 by correcting *two bounded magnitude* substitution errors. Note that the sequence of the homopolymer symbols is known from the consensus.

## System Implementation

Because we tested address-based DNA data storage methods for ordinary text files^4^, for practical implementation we focused on image data. Two images were used as test samples: A poster for the movie Citizen Kane (released in 1941), and a color Smiley Face emoji. The total size of the images was 10,894 bytes. The two images were compressed into a JPEG^17^ format and then converted into a binary string using *Base64*^18^ (*Base64* allows one to embed images into HTML files). The resulting size for the two compressed images was 3,633 bytes.

Through the previously described data encoding methods, the images were converted into 17 DNA blocks, out of which 16 blocks were of length 1,000 bp and one single block was of length 880 bp. Before the sequences were submitted for synthesis, they were tested by the IDT (Integrated DNA Technologies) gBlocks® Gene Fragments Entry online software; they were then synthesized. The total cost of the testing and synthesis was $2,540. IDT failed to synthesize one of the blocks because of a high GC-content in one substring of the address sequence, which was subsequently corrected through the addition of adapters at the two ends of the sequences. Based on information about this type of synthesis error, the sequence encoding procedure was modified to accommodate balancing of all short substrings of the DNA blocks, including the addresses, as previously described. This reduced the synthesis error rate and synthesis time.

The gBlocks representing our DNA codewords synthesized by IDT were mixed in equimolar concentration. One microgram of pooled gBlocks was used to construct the Oxford Nanopore libraries with the Nanopore Sequencing kit SQK-MAP006. The gBlock libraries were pooled and sequenced for 24 hours in a *portable size* MinION MkI using R7 chemistry and flowcell Mk 1 FLO-MAP103.12 with sequencing speed ~75 bp/s. All of the reads used in our subsequent testing were generated within the first 12 hours of sequencing. Base-calling was performed in real time with the cloud service of Metrichor (Oxford, UK); the run produced a total of 6,660 reads that passed the filter. Table 2 provides a summary of the alignment results for all obtained reads, with respect to the reference genomes, along with the types of errors observed. It also illustrates how our new consensus formation algorithm significantly outperforms nanopolish. After the consensus formation stage, the error rate reduced to a mere 0.02% without any error-correction redundancy. It is important to observe that there are two levels of errors we are dealing with: per read and per consensus errors. Sequencing coverage clearly allows for the consensus error to be significantly smaller than the average per read error.

**Table 2:** Summary of the readout data, along with the number and type of errors encountered in the reads.

Block (length)	Number of reads	Sequencing Coverage depth		Number of errors: (substitution, insertion, deletion)		
		Average	Maximum	Per read (average)	Consensus	
					Nanopolish	Our method
1 (1,000)	201	176.145	192	(107, 14, 63)	(14, 32, 5)	(0, 0, 2)
2 (1,000)	407	315.521	349	(123, 12, 70)	(75, 99, 40)	(0, 0, 0)
3 (1,000)	490	460.375	482	(80, 23, 42)	(10, 45, 0)	(0, 0, 0)
4 (1,000)	100	81.763	87	(69, 18, 37)	(1, 54, 1)	(0, 0, 0)
5 (1,000)	728	688.663	716	(88, 20, 48)	(4, 45, 3)	(0, 0, 0)
6 (1,000)	136	120.907	129	(79, 21, 42)	(390, 102, 61)	(0, 0, 0)
7 (1,000)	577	542.78	566	(83, 26, 41)	(3, 31, 3)	(0, 0, 0)
8 (1,000)	217	199.018	207	(83, 20, 46)	(18, 51, 1)	(0, 0, 0)
9 (1,000)	86	56.828	75	(60, 16, 30)	(404, 92, 54)	(0, 0, 0)
10 (1,000)	442	396.742	427	(91, 18, 52)	(388, 100, 59)	(0, 0, 0)
11 (1,000)	114	101.826	110	(79, 23, 42)	(16, 23, 18)	(0, 0, 0)
12 (1,000)	174	162.559	169	(94, 23, 50)	(14, 59, 1)	(0, 0, 0)
13 (1,060)	378	352.35	366	(88, 26, 44)	(7, 55, 4)	(0, 0, 0)
14 (1,000)	222	189.918	203	(69, 22, 34)	(15, 34, 3)	(0, 0, 0)
15 (1,000)	236	222.967	232	(92, 24, 45)	(15, 46, 2)	(0, 0, 0)
16 (1,000)	198	182.99	195	(103, 16, 61)	(15, 62, 4)	(0, 0, 1)
17 (880)	254	240.273	250	(77, 19, 42)	(359, 95, 44)	(0, 0, 0)

**Figure 3:**
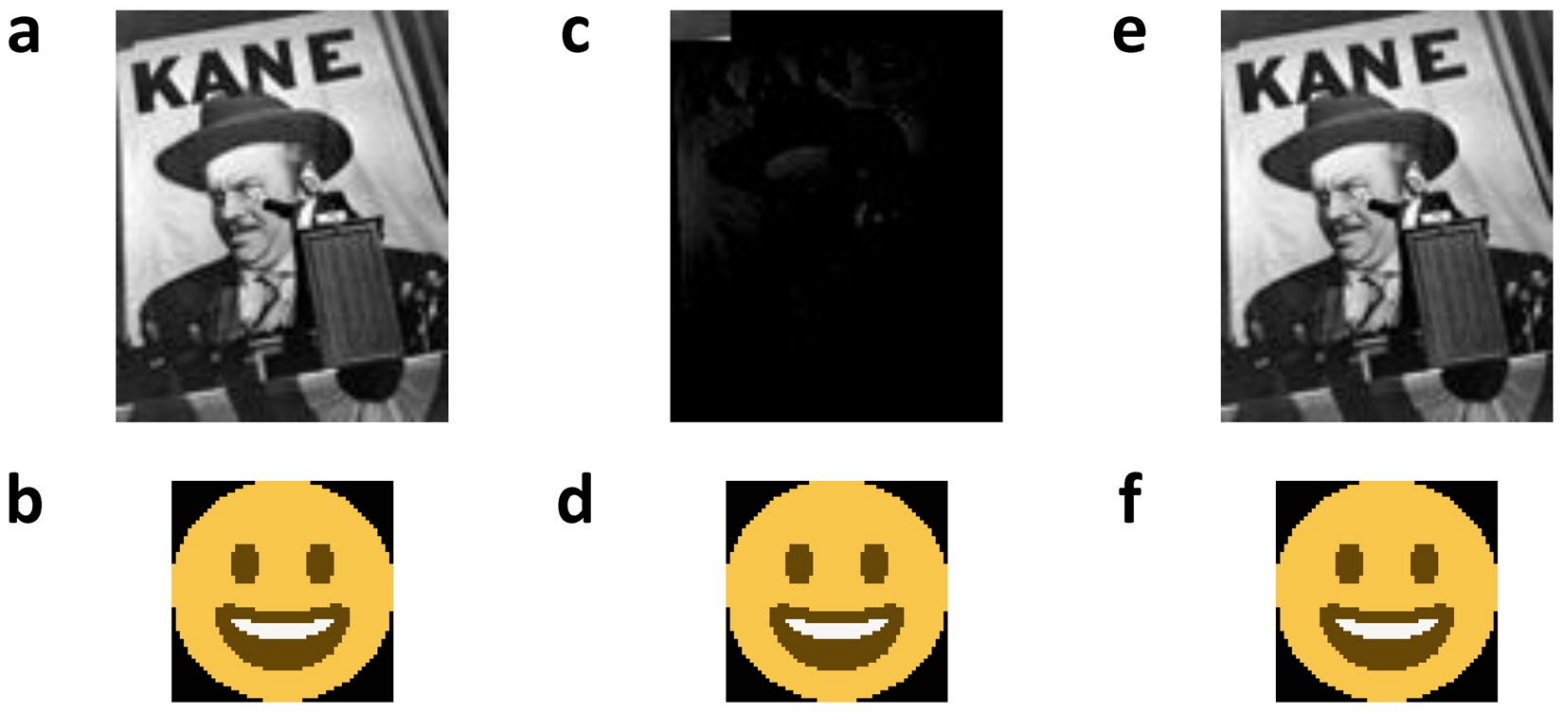
Image files used in our experiment. (**a**,**b**) show the raw images which were compressed, encoded and synthesized into DNA blocks. The Citizen Kane poster^19^ (photographed by Kahle, A., date of access: 17/11/2016) RKO Radio Pictures, not copyrighted per claim of Wikipedia repository) and Smiley Face emoji were of size 9,592 and 130.2 bytes, and had dimensions of 88 × 109 and 56 × 56 pixels, respectively. (**c**,**d**) show the recovered images after sequencing of the DNA blocks and the post-processing phase without homopolymer error correction. Despite having only two errors in the Citizen Kane file, we were not able to recover any detail in the image. On the other hand, one error in the Smiley Face emoji did not cause any visible distortion. (**e**,**f**) show the image files obtained after homopolymer error correction, leading to an error-free reconstruction of the original file.

The three residual errors in the 17 consensus codewords were of the following type: in one block, two homopolymers AAAAAAA were erroneously decoded to AAAAA, while in one block, the homopolymer AAAAA was converted into AAAA. Error patterns where long homopolymer lengths are being reduced by one or two were also observed in the raw reads, as well as in other experiments that we will report on elsewhere. These asymmetric homopolymer errors were subsequently corrected using homopolymer checks, thereby producing error-free reconstructed images. The images reconstructed with and without homopolymer checks are shown in Fig. 3 (e,f) and Fig. 3 (c,d), respectively.

The described implementation represents the only known random access DNA storage system that operates in conjunction with a MinION sequencer. Despite the fact that MinION has significantly higher error rates than Illumina sequencers and that random-access DNA systems typically require additional data redundancy, our DNA storage system has the highest reported information rate of 0.85, storage density of $4\,\times \,{10}^{20}$ bytes/gram, and it offers error-free reconstruction.

### Data availability

The sequencing data are available at Google Drive: https://drive.google.com/open?id=0BwIM8p8qEKCaU1NlRzFWTjltZ2M.

### Software availability

The encoding, alignment and decoding algorithms are available at GitHub: https://github.com/smhty/MATLAB_MinION.

## Electronic supplementary material

Supplementary Information
