# Author Correction: Portable and Error-Free DNA-Based Data Storage

**DOI:** 10.1038/s41598-020-60080-9

**Published:** 2020-04-22

**Authors:** S. M. Hossein Tabatabaei Yazdi, Ryan Gabrys, Olgica Milenkovic

**Affiliations:** University of Illinois, Department of Electrical and Computer Engineering, Urbana, 61801 United States

Correction to: *Scientific Reports* 10.1038/s41598-017-05188-1, published online 10 July 2017

Expression (9) in the Supplementary Information contains two typographical errors. The multiplier 0.85 should be removed from the numerator, while the denominator should include the term 650/2 to account for the average mass of a nucleotide (in daltons). When corrected, the storage density equals 4×10^20^ bytes/gram.

Therefore, the text in the Abstract,

“… while still producing error-free readouts with the highest reported information rate/density.”

now reads:

“… while still producing error-free readouts with the highest reported information rate.”

In addition, the text in the final paragraph of the section ‘System Implementation’,

“… our DNA storage system has the highest reported information rate of 0.85, storage density of 1.1 × 10^23^ bytes/gram, and it offers error-free reconstruction.”

now reads:

“… our DNA storage system has the highest reported information rate of 0.85, storage density of 4 × 10^20^ bytes/gram, and it offers error-free reconstruction.”

These errors have now been corrected in the HTML and PDF versions of this Article, and in the accompanying Supplemental Material.

