# Supplementary material for: Portable and Error-Free DNA-Based Data Storage
Source: Sci Rep. 2017 Jul 10;7:5011. doi: 10.1038/s41598-017-05188-1 (PMC5503945; doi:10.1038/s41598-017-05188-1)
Supplement: Supplementary file 1 — Supplementary Information [file 41598_2017_5188_MOESM1_ESM.pdf]

Supplementary Information
Portable and Error-Free DNA-Based Data Storage

S. M. Hossein Tabatabaei Yazdi[1], Ryan Gabrys[1] & Olgica Milenkovic[1,*]

[1]University of Illinois, Department of Electrical and Computer Engineering, Urbana, 61801, U. S.
*milenkov@illinois.edu

Before data is encoded into DNA, it is compressed to reduce the file size and hence lower the cost of DNA synthesis. Any compression method is compatible with our encoding procedures and is left as a choice for the user. Given that the focus of this study is image storage, the compression method used throughout our analysis and implementation is JPEG. The actual process of encoding refers to converting the compressed data into DNA codewords of length 1,000 bp (gBlocks). Note that we alternatively refer to the codewords as DNA blocks or gBlocks.

Despite a large amount of work on oligo-based data storage, we used gBlocks as they have many advantages over oligo sequences, including:

1. Long DNA sequences may be easily assembled with minimal or no coverage redundancy even when no addressing schemes are used. When using addresses, one effectively mitigates the need for assembly if the address sequences are carefully designed. Furthermore, for long codeword lengths, the overhead introduced by address sequences is negligible, as the length of the address is typically logarithmic in the length of the codeword.
2. Long DNA sequences offer largest coding efficiency as the address overhead for oligos may be as large as 20%, while for gBlocks it amounts to a mere 2%. Furthermore, all known coding schemes offer best performance for long block lengths.
3. Most third-generation sequencing technologies are being developed to accommodate long reads, and hence long-read sequencers will become the dominant market product in the near future; at the same time, a number of companies (e.g., IDT) offer smallest costs per base pair for long DNA sequences (for IDT, this cost equals 14 cents). Note that currently, a significantly lower cost for gBlocks may be obtained through Gen9 (https://www.gen9bio.com).

All modern storage systems offer random access, as otherwise one would have to retrieve the complete data content to read out even one bit of desired information. As every random-access data storage system is built around addressing schemes, we start with a description of address sequence construction and encoding. The results presented in the next section build upon our previous random-access architecture[1], with the important difference that this work presents the first explicit mathematical construction of exponentially large sets of addresses.

Data Encoding. We represent a DNA codeword (block) by a row vector over the four symbols alphabet $\mathcal{D} = \{\mathrm{A}, \mathrm{C}, \mathrm{G}, \mathrm{T}\}$. Moreover, we make frequent use of the following notation:

- If $\mathbf{a} = (a_1, \cdots, a_n)$ and $\mathbf{b} = (b_1, \cdots, b_m)$ are two vectors over the same alphabet $\mathcal{D}$, then $\mathbf{ab}$ is the vector obtained by appending $\mathbf{b}$ to $\mathbf{a}$, i.e., $\mathbf{ab} = (a_1, \cdots, a_n, b_1, \cdots, b_m)$.
- If $\mathbf{a} = (a_1, \cdots, a_n)$, then $\mathbf{a}_i^j = (a_i, \cdots, a_j)$ is used to denote the substring of $\mathbf{a}$ starting at position i and ending at position j, $1 \leq i \leq j \leq n$.
- If $\mathbf{a} = (a_1, \cdots, a_n)$, then $\mathbf{a}^{(i)} = \mathbf{a}_i^n \mathbf{a}_1^{i-1}$ is the cyclic shift of the vector $\mathbf{a}$ starting at position $1 \leq i \leq n$. Note that for the shift to be well defined we impose the initial condition $\mathbf{a}^{(1)} = \mathbf{a}$.

Next, we describe the encoding scheme used for constructing DNA codewords that may contain arbitrary user information. Each DNA block starts with a *unique address* of length p base pairs; the remaining symbols in the block are referred to as the encoded information part of the block. Using the previously introduced notation we may write each DNA block as $\mathbf{ab}$, where $\mathbf{a}$ denotes the address and $\mathbf{b}$ denotes the encoded information part. We also denote the set of all the allowed address sequences of length p and all valid encoded information sequences of length N-p by $\mathcal{A}$ and $\mathcal{B}$, respectively. Hence, $\mathbf{a} \in \mathcal{A}$ and $\mathbf{b} \in \mathcal{B}$ and the total codelength equals N.

The encoding goals are three-fold. The first goal is to design a set of addresses and encoded information sequences such that for any two not necessarily distinct addresses $\mathbf{a}, \mathbf{a}' \in \mathcal{A}$, and for any encoded information sequence $\mathbf{b} \in \mathcal{B}$, $\mathbf{a}'$ does not appear as a substring anywhere in $\mathbf{a}_2^p \boldsymbol{b}$. In other words, if $\mathbf{c} = \mathbf{ab}$ then $\mathbf{a}' \neq \mathbf{c}_i^{p+i-1}$, for $i \geq 2$. The second goal is to make the addresses as distinguishable as possible, and ideally the solution for this problem would be to use sequences at large edit distance (Recall that the edit distance between two strings equals the smallest number of deletions, insertions and substitutions needed to convert one string into the other). Working with the edit distance causes a number of mathematical challenges, and we instead adopt an approach that requires the Hamming distance between pairs of different addresses to be as large as possible (The Hamming distance between two sequences $\mathbf{a}, \mathbf{a}' \in \mathcal{A}$ is defined as $d_H(\mathbf{a}, \mathbf{a}') = |\{i: a_i \neq b_i\}|$). The third goal is to balance out the GC-content of sequences in $\mathcal{B}$ locally. Local balancing refers to balancing the GC-content in each relatively short substring of the DNA codewords. Local balancing simplifies the process of synthesis, enables error correction and makes rewriting easier to accommodate with short primers. These properties will be discussed further in the experimental study section.
More formally, with respect to the first design goal, our address sequences are required to satisfy the following property.
Property 1. *For any two not necessarily distinct addresses* $\boldsymbol{a}, \boldsymbol{a}' \in \mathcal{A}$, *and for any encoded information sequence* $\boldsymbol{b} \in \mathcal{B}$, $\boldsymbol{a}'$ *is not allowed to appear as a substring in* $\boldsymbol{a}_2^p \boldsymbol{b}$, *i.e.,* $\boldsymbol{a}' \neq (\boldsymbol{ab})_i^{p+i-1}$, *for* $i \geq 2$.
We now describe a simple new construction for sequences that satisfy Property 1. Another sequence family with related properties was introduced in our companion paper[2]. Given a binary set (code) $\mathcal{C} \subseteq \{0,1\}^n$, let $\mathcal{C}^{\text{cyclic}}$ denote the set of all cyclic shifts of elements in $\mathcal{C}$, that is if $\boldsymbol{a} \in \mathcal{C}$ then $\boldsymbol{a}^{(i)} \in \mathcal{C}^{\text{cyclic}}$, for $1 \leq i \leq n$. Consider two binary codes $\mathcal{C}_1, \mathcal{C}_2 \subseteq \{0,1\}^n$ such that

- $\mathcal{C}_1^{\text{cyclic}} \cap \mathcal{C}_2 = \emptyset$.
- If $\mathbf{a} \in \mathcal{C}_1$, then $\mathbf{a}^{(i)} \notin \mathcal{C}_1$ for $2 \leq i \leq n$.

Given these two binary codes, we first construct the set of addresses $\mathcal{A} \subseteq \{\mathrm{A}, \mathrm{C}, \mathrm{G}, \mathrm{T}\}^{2n}$ according to:

$$\mathcal{A} = \{\psi(\mathbf{ff}, \mathbf{g}) | \mathbf{f} \in \mathcal{C}_1, \mathbf{g} \in \mathcal{C}_3\}. \quad (1)$$

Note that the address length is $p = 2n$, $\mathcal{C}_3$ is a binary code of length $p = 2n$ whose properties may be chosen so as to enforce a minimum Hamming distance on the addresses, and $\psi(\cdot,\cdot)$ is a bijection that maps two binary strings into one DNA codeword. More precisely, if $\mathbf{f} = (f_1, \cdots, f_m) \in \{0,1\}^m$ and $\mathbf{g} = (g_1, \cdots, g_m) \in \{0,1\}^m$, then $\psi(\mathbf{f}, \mathbf{g}) = \mathbf{h} \in \{\mathrm{A}, \mathrm{C}, \mathrm{G}, \mathrm{T}\}^m$, where $\mathbf{h} = (h_1, \cdots, h_m)$ is obtained according to the rules:

$$h_i = \begin{cases} \mathrm{A}, & \text{if } (f_i, g_i) = (0,0) \\ \mathrm{T}, & \text{if } (f_i, g_i) = (0,1) \\ \mathrm{C}, & \text{if } (f_i, g_i) = (1,0)' \\ \mathrm{G}, & \text{if } (f_i, g_i) = (1,1) \end{cases} \quad \text{for } 1 \leq i \leq m. \quad (2)$$

The set of encoded information sequences $\mathcal{B}$ is defined as a collection of DNA strings of the form

$$\mathcal{B} = \{\mathbf{s}_1 \cdots \mathbf{s}_m | \text{for } m \geq 1, \mathbf{s}_i \in \mathcal{S}\}, \quad (3)$$

where the set $\mathcal{S}$ equals

$$\mathcal{S} = \{\psi(\mathbf{f}, \mathbf{g}) | \mathbf{f} \in \mathcal{C}_2, \mathbf{g} \in \mathcal{C}_4\}. \quad (4)$$

Here $\mathcal{C}_2, \mathcal{C}_4$ are binary code of length n whose properties may be tuned to accommodate balancing and Hamming distance constraints.
The next theorem shows that the sequences in $\mathcal{A}$ and $\mathcal{B}$ satisfy Property 1.
Theorem 1. *$\mathcal{A}$ and $\mathcal{B}$ defined in 1 and 3, satisfy Property 1.*

Proof. Consider two arbitrary address sequences $\boldsymbol{a}, \boldsymbol{a}' \in \mathcal{A}$ and an encoded information sequence $\mathbf{b} \in \mathcal{B}$. For simplicity of notation assume that $\mathbf{a} = \psi(\mathbf{ff}, \mathbf{g})$, $\mathbf{a}' = \psi(\mathbf{f}'\mathbf{f}', \mathbf{g}')$ and that $\mathbf{b} = \mathbf{s}_1 \cdots \mathbf{s}_m$, where $\mathbf{s}_i = \psi(\mathbf{f}_i, \mathbf{g}_i)$ for $1 \leq i \leq m$. Since the mapping $\psi(\cdot,\cdot)$ defined above is one-to-one, the claimed result will follow if we can prove that $\mathbf{f}'\mathbf{f}'$ does not appear as a substring anywhere in $\mathbf{f}_2^n \mathbf{f} \mathbf{f}_1 \cdots \mathbf{f}_m$. This can be done by checking two conditions:

- $\mathbf{f}'$ does not appear as a substring anywhere in $\mathbf{f}_2^n\mathbf{f}$**.** Otherwise, $\mathbf{f}'$ would have to be a proper cyclic shift of $\mathbf{f}$, i.e., there would exist an index $2 \leq i \leq n$ such that $\mathbf{f}' = \mathbf{f}^{(i)}$. But then $\mathbf{f}' = \mathbf{f}^{(i)} \notin \mathcal{C}_1$, which contradicts the assumption that $\mathbf{f}, \mathbf{f}' \in \mathcal{C}_1$.
- $\mathbf{f}'\mathbf{f}'$ does not appear as a substring in $\mathbf{f}\mathbf{f}_1 \cdots \mathbf{f}_m$. Otherwise, there would exist a sequence $\mathbf{f}_i$ for $1 \leq i \leq m$ that appears as a substring in $\mathbf{f}'\mathbf{f}'$. This would in turn imply that $\mathbf{f}_i$ is a cyclic shift of $\mathbf{f}'$, i.e., $\mathbf{f}_i \in \mathcal{C}_1^{\text{cyclic}}$. This contradicts our initial assumptions that $\mathbf{f}_i \in \mathcal{C}_2$ and $\mathcal{C}_1^{\text{cyclic}} \cap \mathcal{C}_2 = \emptyset$. ■

We now turn our attention to the practical implementation of this scheme and the choice of various code components and parameters. In our experiments, we set $n = 8$ and designed addresses of length $p = 16$. We also selected $\mathcal{C}_2$ and $\mathcal{C}_4$ to be the set of all balanced binary words (i.e., words with half 0s and half 1s) and all binary words of length 8, respectively. Note that $|\mathcal{C}_2| = \binom{8}{4}, |\mathcal{C}_4| = 2^8$. In addition, according to the defining relation (2), $\psi(\mathbf{f}, \mathbf{g})$ is a GC-balanced DNA string if and only if $\mathbf{f}$ is a balanced binary word. So, since $\mathcal{C}_2$ is a balanced set, $\mathcal{S}$ is just the set of all GC-balanced DNA strings of length 8 bp. The cardinality of the latter set equals $|\mathcal{S}| = \binom{8}{4} \times 2^8$, and $\mathcal{B}$ is formed by stringing together elements from $\mathcal{S}$. An important observation which will subsequently be used in our post-processing step is that balancing the GC-content of each substring of a given length limits the longest homopolymer length to the same value – in our case, 8.

To construct a large set of addresses, one may select $\mathcal{C}_1 = \{10000000, 01111111\}$ and let $\mathcal{C}_3$ be the set of all binary strings of length 16. In this case, the number of addresses equals $|\mathcal{A}| = 2^{17}$. Alternatively, one may select the code $\mathcal{C}_3$ to have large Hamming distance which would result in the same Hamming distance for the constructed addresses (in our experiments, we used an extended [16,11,4] BCH code for $\mathcal{C}_3$). It is easy to verify that in this case $\mathcal{A}$ and $\mathcal{B}$ satisfy the condition of Property 1. Also of importance are the numerically computed Hamming distances between the chosen addresses of the blocks and all the substrings of the encoded DNA blocks of the same length. For each address of length 16 we hence recorded the distance between the address and all the substrings in the codewords. We then identified the "most similar" substrings for the address sequences in terms of the Hamming distance and replaced the later if needed to achieve larger discriminative power during PCR reactions.

Using the described method, we designed 17 DNA blocks, each of length 1,000 bp, containing 984 bp of encoded information involving a black and white movie poster (Citizen Kane)[3] and a color image (Smiley Face). Here, $\mathcal{B}$ was formed by grouping together 123 balanced strings from $\mathcal{S}$. The block addresses are listed in the Extended Data Table 1, along with the average and minimum Hamming distances between our chosen addresses and encoded substrings. Note that the choice of the BCH code $\mathcal{C}_3$ is justified by the fact that the minimum Hamming distance between a DNA address and any encoded information substring equals $d_H = 4$.

DNA Synthesis. The blocks constructed using the binary component codes were synthesized by Integrated DNA Technology (IDT). Before performing the actual writing process, the synthesis complexity of each DNA codeword was tested by a special purpose IDT software (http://www.idtdna.com/pages/products/genes/gblocks-gene-fragments). It appears that synthesis errors are highly correlated with the total repeat density (direct, inverse and palindromic repeat elements), extreme variations in GC-content, and secondary structures, especially if such properties hold near the 3' and 5' termini of the sequence. All these issues were accommodated to the largest extent possible via our balancing and address selection procedures.

Although all the 17 DNA blocks passed the initial complexity test, IDT was not able to synthesize one of the blocks due to high GC-content in one substring of the corresponding address sequence (See Extended Data Figure 1 (a)). To overcome the problem, we redesigned the address and in addition, requested terminal adapters to be added to the original DNA block in order to check if the hard-to-synthesize sequence had other undesirable properties that may arise only during sequencing. The sequences augmented by adapters passed all subsequent tests without issues. Note that IDT maintains a small subset of adapters which have been optimized to be compatible with the gBlock synthesis process (See Extended Data Figure 1 (b)). These adapters can be appended to the 5' and 3' ends of any sequence and may increase synthesis feasibility whenever complex secondary structures are encountered.

DNA Sequencing. Nanopore sequencers are built around nanopores, small holes with an internal diameter of the order of 1 nm. The idea behind nanopore sequencing is that when a nanopore is immersed in a conducting fluid and a voltage is applied across it, an electric current due to conduction of ions through the nanopore is induced. The current is very sensitive to the size and shape of the nanopore. If DNA strands or other molecules pass through the nanopore, they create a characteristic change in the magnitude of the current through the nanopore, and one can use the generated signal and sequence the DNA strands[4]. MinION is the first commercial sequencer using nanopore technology, and it was released by Oxford Nanopore Technologies in 2014. It is also very small compared to the other sequencing devices available, 10×3×2 cm in size and it weighs just 90 g. DNA sequencing on MinION is performed by first adding the sample to the flowcell that contains all the nanopores that are needed to perform sequencing. When DNA strings pass through nanopores, there will be a change in electrical current, and this current in the nanopore is measured and sampled by a sensor several thousand times per second. Base-calling is performed on 5-mers or 6-mers[5]. In our experiments, we used the R7 chemistry, with a throughput of at least 75 base pairs/s. The latest model, R9, may only improve the system performance compared to R7, as it produces single strand reads, deep-learning base calling, and simpler workflows. Still, none of these features were critical for demonstrating the power of our coding schemes. The readouts of R9 are also created with smaller delay, but yet again, the delay parameter does not influence our findings.

Random Access (RA). RA is performed using PCR reactions with primers corresponding to the addresses of the selected blocks. The protocols for RA experiments were described in detail in[1].

Reconstructing Sequences from Traces. The problem of reconstructing a sequence from a number of reads that were generated by passing the same sequence multiple times through different "versions" of the nanopore requires using specialized tools from bioinformatics and theoretical computer science. Here, a version may refer to a nanopore used at a certain time or a different nanopore. As Oxford Nanopore MinION uses a biological pore, the more the pore is used, the more likely that the quality of the reads will differ. It is currently not known if the system relies on one or multiple pores to perform sequencing. The main challenge is to identify which pores gave unacceptable quality readouts and perform accurate sequence estimation based on high quality reads only.

Variants of the latter problem have been studied in the computer science literature under the name of *sequence reconstruction via traces* (Extended Data Figure 2). The general traces problem may be stated as follows: Reconstruct a string $\mathbf{x}$ of length n from a set of m subsequences generated by randomly editing symbols in x with a given probability fixed for all subsequences[6]. In a separate work[7], the authors showed that by focusing on edits of the form of deletions, which occur with a small constant probability, $m = poly(n)$ traces suffice for exact reconstruction. Here, $poly(n)$ stands for a polynomial expression in n. Later, this result was improved[8] to show that for certain alphabet sizes, even a sub-polynomial number of traces suffices. Both lines of work also described several algorithms for high probability, error-free reconstruction. Other works[9,10] considered the reconstruction problem for the case that the traces represent nanopore DNA reads. In[9], the authors studied the noiseless setup where each read has some fixed length and a noisy setup in which the reads were subjected to substitution errors. In[10], the authors consider a different type of noise model that more realistically captures the properties of nanopore sequencing technologies. In particular, they gave bounds on the parameter m necessary for reliable reconstruction for the case that edits change the homopolymer lengths. Another related line of work includes[11], where accurate sequence reconstructing via new profile coding techniques was proposed. And although the model studied in[10] is relevant to our approach, it cannot be applied directly due to the fact that the pores introduce a complex mixture of errors that are not necessarily confined to homopolymer changes. In addition, the noise parameters of the pore(s) observed during different readout times may be very different, and they depend on the particular sequence structure as well. Hence, we propose a two-stage procedure that will allow us to mitigate all the aforementioned problems:

Stage 1. Consensus Sequence Formation via Iterative Alignment. Given the different read qualities, it is important to first identify reads with small fractions of deletion and insertion errors which will be used in the first step of reconstruction. For this purpose, we use the idea of *pilot sequences*. A pilot sequence is used to

assess the performance of a noisy channel (e.g., wireless channel) as it is known both to the transmitter and receiver. The transmitter sends the pilot sequence to the receiver which can based on the knowledge of the sequence estimate the probability of error. If the error probability is small, the transmitter asks for the information sequence to be transmitted; otherwise, the transmitter delays transmission. Clearly, as the addresses of the sequences of the DNA codewords are known to the user, they may serve the role of pilot sequences. More precisely, based on the quality of the address sequence one may decide to use a read in the reconstruction process or not. Once good quality reads are identified, the obvious next step is to perform alignment of the reads and then form a consensus sequence through majority voting at each sequence coordinate or for each homopolymer. This procedure may be performed in multiple rounds, using more and more reads to correct consensus errors as needed, especially in areas where the consensus has low confidence values (See the illustrative region of poor sequence alignment in Extended Data Figure 3, obtained using the Tablet Software[12]). Furthermore, as pointed out in the main text, the parameters of various MSA algorithms need to be carefully tuned to produce the best alignment results, and are shown in the Extended Data Table 2.

To better explain our iterative scheme for finding the consensus sequence using majority rules and the balancing property, we provide a simple example. This example involves three reads and the rough estimate of the DNA block after running the first alignment phase described in the main text. The three reads (read1, read2 and read3) and the running consensus estimate (Cest) are listed below:

Cest TTCACCCAAAAACCCGAAAACCGCTTCAGCGA
read1 TTCACCCCAAAACCGAAAACCGCTTCACGA
read2 TTCACCCAAAAACCCGAAAACCGCTTCAGCGA
read3 TTCACCCAAAAACCCGAAAACCGCTTCAGCGA

By running the MATLAB built-in MSA algorithm[13] we obtain the following result:

Cest	TT	C	A	CCCC	AAAA∗	CCC	G	AAAA	CC	G	C	TT	C	A	∗	C	G	A
read1	TT	C	A	CCC∗	AAAA∗	CC∗	G	AAAA	CC	G	C	TT	C	A	∗	C	G	A
read2	TT	C	A	CCCC	AAAA∗	CCC	G	AAAA	CC	G	C	TT	C	A	G	C	G	A
read3	TT	C	A	CCC∗	AAAAA	CCC	G	AAAA	CC	G	C	TT	C	A	G	C	G	A
	r_1	r_2	r_3	r_4	r_5	r_6	r_7	r_8	r_9	r_{10}	r_{11}	r_{12}	r_{13}	r_{14}	r_{15}	r_{16}	r_{17}	r_{18}

We next divide the alignment into 18 nonoverlapping segments corresponding to different maximal length homopolymers. The segments may have different lengths, but due to the balancing constraint, no segment has length exceeding 8. To improve the current estimate Cest, we start by forming the consensus from the left and by adding a new homopolymer at each step. Since for the first three segments all four sequences agree on the homopolymer lengths and do not violate the balancing property, we initiate the new consensus sequence to

cns TTCA.

Next, we add one more homopolymer corresponding to the forth segment (r_4). According to the four sequences, this homopolymer should have length either 3 or 4 (the sequences in question are CCC or CCCC). Note that the majority rule suggests that the correct sequence is CCC; this sequence also satisfies the balancing property, since we know that the next symbol is from the fifth segment and equals A. The second option CCCC does not satisfy the balancing property. Hence, the only valid solution up until this point reads as

cns TTCACCC.

For the fifth segment, the majority rule suggests picking the sequence AAAA as the next homopolymer candidate. Also, it is apparent that we need to have at least four G or C symbols in both the segments r_6 and r_7, as otherwise the resulting block does not have a balanced GC-content. The only homopolymer choices that satisfy these constraints are CCC for r_6 and G for r_7. As all sequences agree on the homopolymer choices for segments r_8 to r_{12}, the 24 symbols of the consensus read as TTCACCCAAAACCCGAAAACCGCT. As may be seen, the last 8 symbols do not have a balanced GC composition. As the first encountered ambiguity in the reads came from segment r_5, we change our homopolymer to AAAAA instead of AAAA, although it violates the majority choice. Then, the consensus up until and including segment 14 equals:

cns TTCACCCAAAAACCCGAAAACCGCTTCA.

(Note that violations of the majority rules as described above are rare, and included only for illustrative purposes.) In the final step, we select the homopolymer G to represent the segment r_{15}, although it again

violates the majority rule - another choice would result in a consensus with 31 symbols, a length not divisible by 8. Hence, the new consensus equals

cns TTCACCCAAAAACCCGAAAACCGCTTCAGCGA

and satisfies the GC-balanced property. This new consensus is used in the BWA reference based alignment software to identify more good and acceptable quality reads that may resolve issues with poor alignment regions. The procedure is repeated until no sufficiently good reads are available, or until all poor alignment regions are resolved or declared unresolvable.

Clearly, one cannot guarantee that the above procedure produces error-free read estimates. But given the choice of the alignment parameters, the majority rules and the balancing checks, one tends to observe only a very small number of errors which tend to be exclusively deletions. In our experiments, we encountered three deletion errors for the whole encoded file: All deletions were confined to homopolymers of length at least five, and exclusively included As. Hence, the information about *homopolymer symbols* was recovered correctly. Extended Data Table 3 (Experiment 1), shows the summary of error events in the DNA blocks after one round of address-anchored alignment and an additional round of BWA alignment.

We conclude by observing that in all alignment procedures, we used less than 200 reads per DNA codeword. Such a small number of reads may be generated in a relatively short time, and it appears to be a rule of thumb that the best quality reads are generated first. Hence, the readout cost and delay of the MinION system are highly competitive with those of other technologies.

We also tested the Nanopolish software[14] (Version 0.6-dev) to obtain the consensus sequences. Nanopolish is a software package for signal-level analysis of Oxford Nanopore sequencing data, and it is used to calculate an improved consensus sequence from an initial draft genome. The commands used to obtain the consensus are as follow:

1. Extract the QC-passed reads from a directory of FAST5 files:

   $ nanopolish extract --type 2d FAST5/ > reads.fa
2. Index the draft genome:

   $ bwa index draft.fa
3. Align the basecalled reads to the draft sequence:

   $ bwa mem -x ont2d -t 8 draft.fa reads.fa | samtools sort -o reads.sorted.bam -T reads.tmp - samtools index reads.sorted.bam
4. Use Nanopolish to compute the consensus sequence:

   $ nanopolish variants –consensus cns.fa –r reads.fa –b reads.sorted.bam –g draft.fa -t 4 -- min-candidate frequency 0.1

To test the performance of Nanopolish, we designed two separate experiments (Extended Data Table 3, Experiment 2 and Experiment 3).

- The first experiment includes two rounds, and follows the same procedure we used during our iterative alignment method. For the first round, we used the 17 known address sequences as pilot sequences and selected 17 DNA blocks from the reads file (reads.fa) to form the draft genomes (draft.fa). We used the draft genome and flowed the Nanopolish workflow to obtain 17 consensus sequences (cns.fa). Next, each consensus sequence was compared to its original DNA block, and the difference were recorded in terms of the number of insertion, deletion, and substitution (Extended Data Table 3, Experiment 2/Round 1).

  The second round repeats the same procedure, with the draft genome being the consensus sequence from the previous round. The result (Extended Data Table 3, Experiment 2/Round 2) suggests that running the Nanopolish software multiple times does not necessarily improve the consensus sequence accuracy.
- In the second experiment, we used the original 17 DNA blocks as the draft genomes and formed the draft.fa. The results (Extended Data Table 3, Experiment 3) suggest that for our data set Nanopolish is

unable to reconstruct the original DNA blocks even in this genie-aided case.

Deletion Correction. There are numerous known coding schemes that can be used to correct standard deletion errors. The principle drawback of all these schemes is their low information rate and the methods not being able to tackle one particular subclass of deletion errors that was encountered in our sequencing experiments.

In what follows, we briefly discuss three prominent techniques from the literature, and review their information rates.

- Watermark coding and variants thereof refer to the process of inserting or super-imposing a known sequence over an information sequence. Typically, the information sequence is then encoded with an outer probabilistic code such as an LDPC (low-density parity-check) code that enables recovery of the encoded message. The decoding proceeds in two stages whereby the first stage makes use of the watermark code to compute likelihoods that are used in the second stage to decode the LDPC code. The watermark codes are typically designed to correct low error rates and these codes have small information rates. For instance, the highest rate code from[15] was 0.71 and this code had a block error rate which exceeded 0.1, even for a raw deletion rate as small as 0.005. A code with a similar information rate was presented in[16], where again the code had a block error rate exceeding 0.1 when the raw deletion rate was below 0.01. Another potential drawback to these codes is their long block length (on the order to several thousand bits long) which may be unsuitable for DNA codewords that are typically of length of the order of 1,000 bp.
- A more recent scheme for correcting deletions was introduced in[17]. The idea is to use a series of error-control codes on patterns that appear in a large majority of the strings. To correct t deletions, in a block of length n the construction from[17] was shown to require $O(t^2(\log t)\ \log n)$ redundant bits; however, for shorter block lengths, the construction requires many redundant bits. For instance, for the case where $t = 2$, it can be shown that this construction requires at least $144 \log_2(n)$ bits of redundancy which renders the approach impractical for shorter block lengths.
- In[18], a number-theoretic code construction was proposed, which extended the congruence for single deletion codes introduced by Levenshtein in[19]. This construction requires many redundant bits especially for longer block lengths. For only $t = 2$, the redundancy (or the number of redundant bits) is linear with respect to the block length n. More specifically, it can be shown that the redundancy (in bits) for the case where $t = 2$ is at least $\log_2 \left\lfloor \frac{1.5^n}{\sqrt{5}} \right\rfloor$ which implies that for $n = 1024$, such a construction would require at least 597 bits of redundancy.

Homopolymer Parity-Check Coding. Motivated by the findings of the iterative anchored alignment phase, we propose a simple deletion correcting code related to the method described in[9] which leverages the so-called "integer sequence" of an information sequence.

To correct t deletions that preserve homopolymer symbols, our encoding scheme requires approximately $t \log_2 N$ bits of redundancy, where N denotes the length of the encoded information sequence. Furthermore, the code partitions the space $\mathbb{F}_4^N$ and hence lends itself to systematic encoding, which is desirable when constructing codes compatible with different nanopore sequencers. We also note that the proposed construction outperforms existing general multiple deletion correcting codes such as those described in[17,18] as it is adapted to the nanopore channel at hand.

We begin by reviewing the notion of an ***integer sequence***. For a vector $x \in \mathbb{F}_4^n$, the integer sequence of x is the sequence of lengths of maximal runs in x. For example, the integer sequence of $x = (0,0,1,3,3,2,1,1)$ is

$$I(x) = (2,1,2,1,2).$$

Similarly, the integer sequence of AATTTGCGAA equals (2,3,1,1,1,2). In the context of DNA coding, we refer to such a sequence as the homopolymer length sequence. We also make use of the ***string sequence*** of a codeword. The string sequence represents the symbols in the maximal runs of x. For example, the string sequence of x equals

$$S(x) = (0,1,3,2,1)$$

since the first run in x has value 0, the next run has value 1 and so on. The string sequence of AATTTGCGAA equals (A,T,G,C,G,A).

It is straightforward to see that one can uniquely recover x given its integer sequence and string sequence. For shorthand, we use $M\big(I(x), S(x)\big)$ to denote the "reconstruction map", a map such that

$$M\big(I(x), S(x)\big) = x.$$

We introduce one more relevant piece of notation. Suppose that $z \in \mathbb{F}_2^N$, i.e., that z is a binary vector of length N. Then, let

$$\mathfrak{B}_t(z) = \{z + e\},$$

where $e \in \{0, -1\}^N$ and e has at most t non-zero components. Given $I, S,$ and $\mathfrak{B}_t$ we are now able to define the types of errors we wish to correct, which we refer to as sticky deletions, a special case of the general family of repetition errors[20].

Let $x \in \mathbb{F}_4^N$. In our model, we assume that $y \in \mathbb{F}_4^{N-s}$ (where $s \leq t$) is such that

1. $S(y) = S(x)$, and
2. $I(y) \in \mathfrak{B}_t\big(I(x)\big)$.

Note that the first condition enforces that deletions occurring in x leading to y can never cause runs to be added or removed. In other words, the deletions are not allowed to change the string sequence. The second restriction enforces that deletions occurring in x can cause each run length in x *to decrease by at most one*.

We define next a code $\mathcal{C}(N, t)$ capable of correcting sticky deletions. For $x \in \mathbb{F}_4^N$, let $|x|$ denote the number of runs in x. We assume in what follows that for $|x| = r$, where $r < N$, the output of $I(x)$ has length N with the last $N - r$ components of $I(x)$ set to zero.

Suppose that $\mathcal{C}_H(N, t)$ is a binary code of length N capable of correcting up to t substitution errors. Let $\mathcal{C}(N, t) \subseteq \mathbb{F}_4^N$ be defined so that

$$\mathcal{C}(N, t) = \{x \in \mathbb{F}_4^N : I(x) \bmod 2 \in \mathcal{C}_H(N, t)\}.$$

Let $\mathcal{D}_t$ denote a decoder for $\mathcal{C}_H(N, t)$ where if $y = z + e^{(2)} \in \mathbb{F}_2^N$, $z \in \mathcal{C}_H(N, t)$, and $e^{(2)} \in \mathbb{F}_2^N$ has at most t non-zero components, then $\mathcal{D}_t(y) = z$. We prove next that the code $\mathcal{C}(N, t)$ can correct up to t sticky deletions.

Theorem 2. *The code $\mathcal{C}(N, t) \subseteq \mathbb{F}_4^N$ can correct up to t sticky deletions.*

Proof: We prove the result by outlining the decoding algorithm. Suppose that the vector $x \in \mathcal{C}(N, t)$ is stored and that the vector $y \in \mathbb{F}_4^{N-s}$ is retrieved, where $s \leq t$ is the result of s sticky deletions.

First, we compute $S = S(y)$. Since y is the result of at most t sticky deletions, we know that $S = S(x)$. We now show how to recover $I(x)$. Since y is the result of at most t sticky deletions occurring to x, $I(y) \in \mathfrak{B}_t(I(x))$, so that we may write $I(y) = I(x) + e$, where e has at most t non-zero components and each component has value -1. Notice that

$$I(y)\ mod\ 2 \equiv (I(x) + e) mod\ 2 \equiv I(x)\ mod\ 2 + e\ mod\ 2$$

where $I(x) \bmod 2 \in \mathcal{C}_H(N, t)$ and e is a binary vector with at most t non-zero components. Therefore, applying $\mathcal{D}_t$ to $I(y)\ mod\ 2$ gives

$$\mathcal{D}_t(I(y) mod\ 2) = \mathcal{D}_t(I(x)\ mod\ 2 + e\ mod\ 2) = I(x)\ mod\ 2.$$

From the previous equation, given $I(x)\ mod\ 2$, we can determine $e\ mod\ 2$. Notice that every non-zero component at position i in $e\ mod\ 2$ is the result of a sticky deletion. Therefore, we increment the value of $I(y)$ at position i by one to obtain $I(x)$. Using the map M we may then reconstruct $x = M(I(x), S)$.■

Note that the construction in Theorem 1 is not systematic. We describe next how to use the scheme to perform systematic encoding. We consider the case where $t = 2$ and use a primitive BCH code over $\mathbb{F}_2$ of length 1023 and dimension 1003, shortened on the last 23 bits. The resulting code has length 1000, dimension 980, and can correct up to 2 random substitution errors. We denote this code by $C(1000,980,2)$. Since $C(1000,980,2)$ is a linear code, there exists a systematic encoder for $\mathcal{C}_H$, which we denote by Enc, that given an input $w \in \mathbb{F}_2^{980}$, outputs 20 parity bits such that $\big(w, Enc(w)\big) \in C(1000,980,2)$.

We encode our information sequence, denoted by $M_4 \in \mathbb{F}_4^{980}$ into a codeword $x \in \mathbb{F}_4^{1000}$ according to the following procedure:

1. We set the first 980 symbols of x to be equal to M_4 and let $= Enc\big(I(M_4)\big) \in \mathbb{F}_2^{20}$.
2. We convert z to a quaternary representation and denote the resulting sequence with $z^{(4)} \in \mathbb{F}_4^{10}$.
3. We set $(x_{981}, x_{982}, x_{983}, \dots, x_{1000}) = (z_1^{(4)}, z_1^{(4)}, z_2^{(4)}, z_2^{(4)}, \dots, z_{10}^{(4)}, z_{10}^{(4)})$.

Note that since z is binary, it follows that 10 symbols over $\mathbb{F}_4$ suffice to store z. Observe also that the last 20 symbols in x arise by simply repeating every symbol in $z^{(4)}$ twice. Hence, it is straightforward to prove the following corollary.

Corollary 1. *Suppose x is encoded according to the previous procedure and y is the result of up to 2 sticky deletions in x. Then, it is possible to recover x from y.*

Proof: Let v equal the last 20 symbols in y read in reverse order. In other words, the first symbol of v equals the last symbol in y, the second symbol in v equals the second to last symbol in y, and so on. Let z_R be equal to the last 20 symbols in x (which results from repeating every symbol in $z^{(4)}$, generated by our encoding procedure) read in reverse order. We show that it is possible to recover z_R from v given that at most 2 sticky deletions occurred in the string. In fact, it can be shown that $\left(z_1^{(4)}, z_1^{(4)}, z_2^{(4)}, z_2^{(4)}, \dots, z_{10}^{(4)}, z_{10}^{(4)}\right)$ can be recovered given any number of sticky deletions. Note that if z_R is known, one can easily recover the parity bits z and combine the parity bits (which have no errors) with the information symbols in x to construct a vector $\hat{y} \in \mathfrak{B}_2(x)$, where $\hat{y}$ has at most 2 sticky deletions in the information symbols and $x \in \mathcal{C}(N,t)$.
Consider the sequences $I(v) = (u_1, u_2, \dots, u_{|v|}), I(z_R) = (s_1, s_2, \dots, s_{|z_R|})$, and $S(v)$. As a consequence of the sticky channel definition, $S(v) = S(z_R)$. Note also that for every symbol $u_i \in I(v)$, we have $u_i \in \{s_i, s_i - 1\}$. As a result of the encoding, $s_i \equiv 0 \bmod 2$. Therefore, we can recover s_i from u_i by setting $u_i = u_i + 1$ if $u_i \equiv 1 \bmod 2$ and setting $u_i = u_i$ otherwise. In this manner, we can recover $I(z_R)$ and determine z_R from $M(I(z_R), S(z_R))$.■

The rate of the homopolymer check codes was 0.98, and it allowed for systematic encoding, which cannot be achieved via simple $(d = 1, k = 6)$ run-length-constrained code of slightly higher rate 0.998. Furthermore, the homopolymer parity checks may be stored on classical media which have negligible small error probabilities to further increase the code rate to 0.98. This comes at almost no additional cost, as the homopolymer checks constitute less than 0.02% of the data volume.

Remark: *Note that by using the integer and symbol sequences one may correct a more general class of sticky errors, including both deletions and insertions. In this context, it suffices to use a classical substitution error-correcting code on the integer sequence. Any asymmetries observed in the deletion or insertion patterns can be handled by using modulo constraints akin to those described in the definition of the code $\mathcal{C}(N,t)$. As asymmetries provide additional side information, one may expect a higher coding rate for modulo constrained codes compared to standard substitution error-correcting codes.*

Information Rate and Data Storage Density. The information rate of a code (also known as the code rate) is the proportion of the stored data that represents useful information (i.e., is non-redundant). That is, if the code rate equals *k/n*, for every *k* bits of useful information, the code generates a total of *n* bits of data, of which *n-k* are redundant. In our data storage system, we have three layers of data encoding. We calculate next the information rate for each layer and multiply the results of the three layers to get the overall information rate.

- In the first stage of encoding, we used GC-balanced DNA fragments of length 8 to store data. Note that the total number of GC-balanced DNA string of length 8 is $\binom{8}{4} \times 2^8$. Hence, the information rate of the balancing code equals

$$R_1 = \frac{\log_2[\binom{8}{4}\times 2^8]}{\log_2 4^8} = 0.8831. \tag{5}$$

  Note that the nominator is the logarithm of the number of bits required to represent each GC-balanced DNA string of length 8.
- In the second stage of encoding, we formed each DNA block of length 1,000 bp by concatenating an

address sequence with the balanced blocks. At this stage, only 984 bp are used to store useful data while the remaining 16 bp are reserved for addressing. Hence, the information rate of this stage equals

$$R_2 = \frac{984}{1{,}000} = 0.984. \quad (6)$$

- The third stage of encoding is homopolymer parity-check coding. Here, the code rate equals

$$R_3 = 0.98. \quad (7)$$

The total information rate is obtained by multiplying the three rates from 5, 6 and 7, and equals

$$\mathrm{R} = \text{ Information rate} = 0.8831 \times 0.984 \times 0.98 \approx 0.85. \quad (8)$$

To calculate the DNA information density of our scheme, we use the fact that average mass of a DNA base pair is 650 Daltons, and the mass of a single DNA base 325 Daltons (1 Dalton equals the mass of a single hydrogen atom, or 1.67×10^{-24} grams[21]). As we mapped 3,633 bytes of compressed data into 17 DNA blocks with total number of 16,880 bp, the DNA information density equals

$$\text{DNA Information density} = \frac{3{,}633\ \text{bytes}}{(16{,}880\ \text{bp}) \times (325\ \times 1.67 \times 10^{-24}\ \text{gram/bp})} \approx 4\ \times 10^{20}\,\frac{\text{bytes}}{\text{gram}}. \quad (9)$$

For fairness of comparison with other methods based on oligo pools we normalized our density expression with the mass of a single base. The above result establishes the highest known achievable information rate of all DNA-based data storage systems, even when taking into account systems that do not use addressing or rely on highly accurate, but large volume Illumina sequencers.
For comparative purposes, we reproduced Table 1 from the main text of this work, now Extended Data Table 4, listing the features of our new storage architecture.

Data availability. The sequencing data are available at Google Drive:
https://drive.google.com/open?id=0BwIM8p8qEKCaU1NlRzFWTjltZ2M

Software availability. The encoding, alignment and decoding algorithms are available at GitHub:
https://github.com/smhty/MATLAB_MinION

References

1. Yazdi, S., Yuan, Y., Ma, J., Zhao, H. & Milenkovic, O. A rewritable, random-access DNA-based storage system. *Sci. Rep.* **5,** 14138 (2015).
2. Yazdi, S., Kiah, H. M. & Milenkovic, O. Weakly mutually uncorrelated codes. *IEEE Int. Symp. Inf. Theory* **2016,** 2649-2653 (2016).
3. File: Citizen-Kane-welles-podium.jpg. https://commons.wikimedia.org/wiki/File:Citizen-Kane-Welles-Podium.jpg (2016).
4. Nanopore sequencing. https://en.wikipedia.org/wiki/Nanopore_sequencing (2017).
5. Lu, H., Giordano, F. & Ning, Z. Oxford nanopore minion sequencing and genome assembly. *Genomics, proteomics & bioinformatics* **14.5,** 265-279 (2016).
6. Batu, T., Kannan, S., Khanna, S. & McGregor, A. Reconstructing strings from random traces. *SODA* ***2004,*** 910-918 (2004).
7. Holenstein, T., Mitzenmacher, M., Panigrahy, R. & Wieder, U. Trace reconstruction with constant deletion probability and related results. *SODA* **2008,** 389-398 (2008).
8. McGregor, A., Price, E. & Vorotnikova, S. Trace reconstruction revisited. *ESA* **2014,** 689-700 (2014).
9. Sok, L., Sole, P. & Tchamkerten, A. Lattice based codes for insertion and deletion channels. *IEEE Int. Symp. Inf. Theory* **2013,** 684-688 (2013).
10. Duda, J., Szpankowski, W. & Grama, A. Fundamental bounds and approaches to sequence reconstruction from nanopore sequencers. Preprint at http://arXiv.org/abs/1601.02420 (2016).
11. Kiah, H. M., Puleo, G. J. & Milenkovic, O. Codes for DNA sequence profiles. *IEEE Trans. Inf. Theory* **62.14,** 3125-3146 (2016).

12. Milne, I. *et al.* Using Tablet for visual exploration of second-generation sequencing data. *Brief. Bioinform.* **14,** 193-202 (2013).
13. MATLAB multialign function. https://www.mathworks.com/help/bioinfo/ref/multialign.html (2016).
14. Nanopolish. https://github.com/jts/nanopolish (2017).
15. Davey, M. C. & MacKay, D. J. C. Reliable communication over channels with insertions, deletions, and substitutions. *IEEE Trans. Inf. Theory* **47.2,** 687-698 (2001).
16. Ratzer, E. Marker codes for channels with insertions and deletions. *Annals of Telecommunications* **60.1,** 29-44 (2005).
17. Brakensiek, J., Guruswami, V. & Zbarsky, S. Efficient low-redundancy codes for correcting multiple deletions. *SODA* **2016,** 1884-1892 (2016).
18. Helberg, A. S. J. & Ferreira, H. C. On multiple insertion/deletion correcting codes. *IEEE Trans. Inf. Theory* **48.1,** 305-308 (2002).
19. Levenshtein, V. I. Binary codes capable of correcting deletions, insertions, and reversals. *Dokl. Akad. Nauk SSSR* **163.4,** 845-848 (1965).
20. Dolecek, L. & Anantharam, V. Repetition error correcting sets: explicit constructions and prefixing methods. *SIDMA* **23,** 2120-2146 (2010).
21. Molecular facts and figures. https://www.idtdna.com/pages/docs/educational-resources/molecular-facts-and-figures.pdf?sfvrsn=4 (2016).
22. Church, G. M., Gao, Y. & Kosuri, S. Next-generation digital information storage in DNA. *Science* **337,** 1628-1628 (2012).
23. Goldman, N. et al. Towards practical, high-capacity, low-maintenance information storage in synthesized DNA. *Nature* **494,** 77-80 (2013).
24. Grass, R. N., Heckel, R., Puddu, M., Paunescu, D. & Stark, W. J. Robust chemical preservation of digital information on DNA in silica with error-correcting codes. *Angew. Chem. Int. Ed.* **54,** 2552–2555 (2015).
25. Bornholt, J. et al. A DNA-based archival storage system. *ASPLOS* **2016,** 637-649 (2016).
26. Erlich, Y. & Zielinski, D. Capacity-approaching DNA storage. Preprint athttp://dx.doi.org/10.1101/074237 (2016).

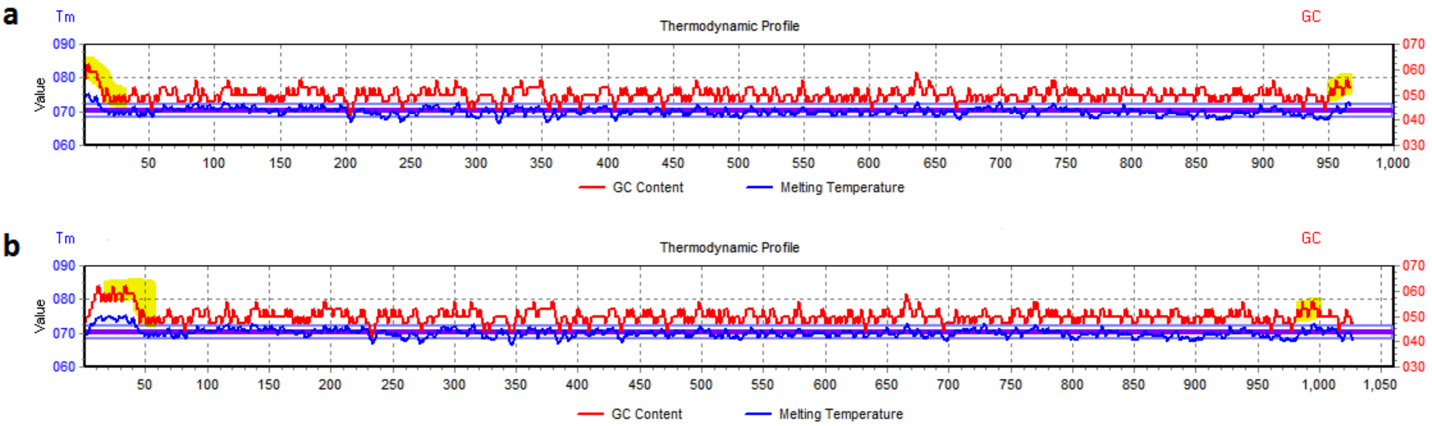

Extended Data Figure 1 | Thermodynamic profiles of sequences with undesirable GC-content. a, The profile of the DNA block with large GC-content in an address substring. **b,** The profile of the sequence upon addition of terminal adapters. It may be observed that the GC-content of the adapters is more balanced that that of the address sequence.

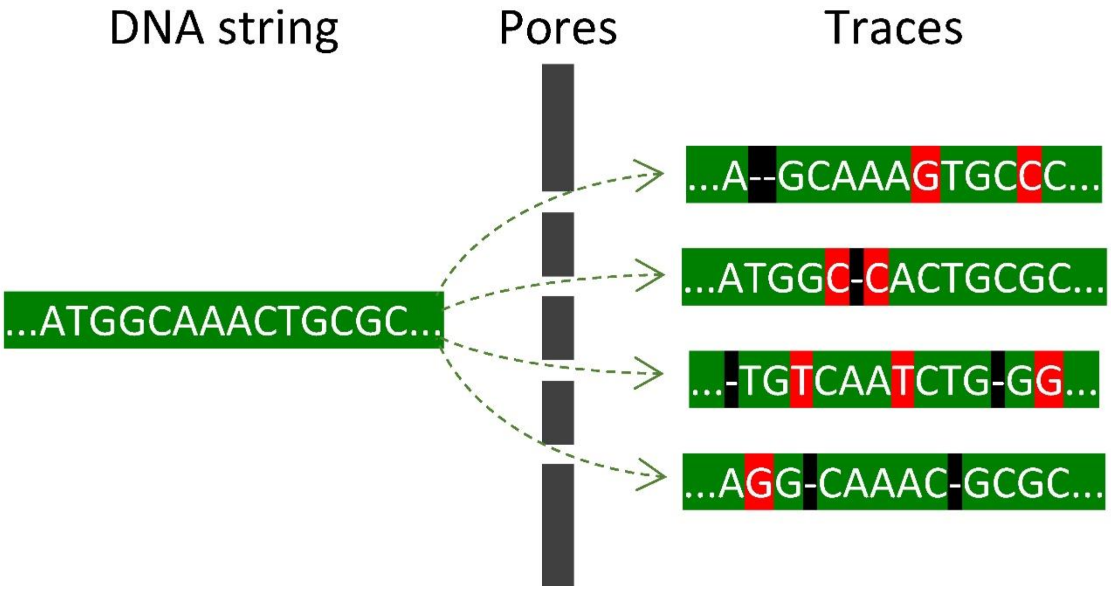

Extended Data Figure 2 | Nanopore-based sequence estimation as a sequence reconstruction from traces problem. A trace refers to the readout of a "pore" at a certain time point, or the readout from one out of multiple pores. During each read time the pore may behave differently, introducing sequence-dependent substitution, deletion and insertion errors at different rates.

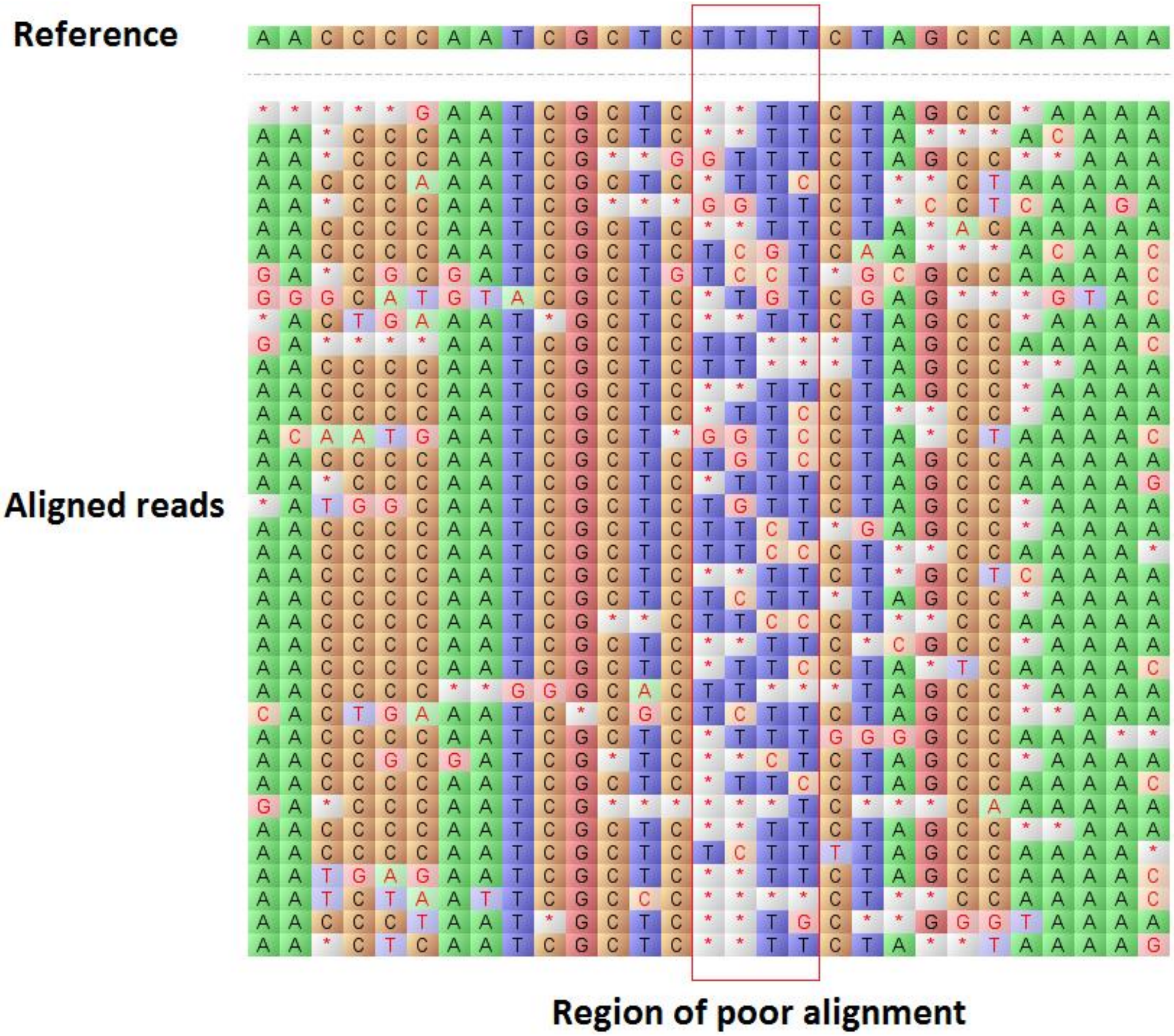

Extended Data Figure 3 | Screen shot from the Tablet software showing poor consensus quality substrings formed during the iterative alignment phase of data recovery.

Block (length)	Average Hamming distance	Minimum Hamming distance	Forward address
**1 (1,000 bp)**	8.75	4	TATGCGCGACCCCCCT
**2 (1,000 bp)**	8.62	4	CCGAATATCAAAAATC
**3 (1,000 bp)**	9.27	5	AATCCGCGACCCCCGA
**4 (1,000 bp)**	9.28	5	CCCAATATCAAAATAG
**5 (1,000 bp)**	9.28	5	AAACCGCGACCCCGCT
**6 (1,000 bp)**	9.34	5	GCCTATATCAAAATTC
**7 (1,000 bp)**	9.30	6	TAAGCGCGACCCCGGA
**8 (1,000 bp)**	9.33	5	CGCAATATCAAATAAC
**9 (1,000 bp)**	9.33	5	ATACCGCGACCCGCCA
**10 (1,000 bp)**	9.32	5	GGCTATATCAAATATG
**11 (1,000 bp)**	9.27	5	TTAGCGCGACCCGCGT
**12 (1,000 bp)**	9.29	5	GGGTATATCAAATTAC
**13 (1,000 bp)**	9.35	4	TTTGCGCGACCCGGCA
**14 (1,000 bp)**	9.2	5	CGGAATATCAAATTTG
**15 (1,000 bp)**	9.2	5	ATTCCGCGACCCGGGT
**16 (1,000 bp)**	9.01	5	AAAGCCCCTGCGCCGT
**17 (880 bp)**	9.23	5	TTACCGCCTCCCCCCA

Extended Data Table 1 | Block addresses and Hamming distance profiles of the addresses vs DNA blocks. Only one address is used.

Software	Parameters
**Kalign**	Gap open penalty = {5,11},<br>Gap extension penalty = {0.2,0.85},<br>Terminal Gap Penalties = {0.1,0.45},<br>Bonus Score = {5.2,5,1,0}
**Clustal Omega**	Number of combined iterations = {0,1,2,3,4,5}
**Coffee**	Default
**MUSCLE**	Default
**MAFFT**	Default
**BWA**	K = 14, W = 20, r = 10, A = 1, B = 1, O = 1, E = 1, L = 0
**MATLAB**	Position of reads in the SAM file is less than or equal to = {1,8,15}

Extended Data Table 2 | Parameter choices for MSA algorithms used to build a consensus of consensus sequences. The parameters are selected to achieve best empirical performance for nanopore read alignments.

Block	Number of errors (substitution, insertion, deletion)				
	Experiment 1		Experiment 2		Experiment 3
	Round 1	Round 2	Round 1	Round 2	
1	(0, 0, 9)	(0, 0, 2)	(14, 36, 8)	(14,32,5)	(0,4,1)
2	(0, 0, 7)	(0, 0, 0)	(83, 93, 58)	(75,99,40)	(3,10,7)
3	(0, 0, 3)	(0, 0, 0)	(11, 48, 1)	(10,45,0)	(0,0,0)
4	(0, 0, 1)	(0, 0, 0)	(4, 57, 2)	(1,54,1)	(0,2,0)
5	(0, 0, 0)	(0, 0, 0)	(8, 52, 10)	(4,45,3)	(0,1,0)
6	(0, 0, 1)	(0, 0, 0)	(397, 100, 54)	(390,102,61)	(0,2,0)
7	(0, 0, 2)	(0, 0, 0)	(8, 34, 4)	(3,31,3)	(0,0,0)
8	(0, 0, 0)	(0, 0, 0)	(22, 53, 2)	(18,51,1)	(1,2,0)
9	(0, 0, 2)	(0, 0, 0)	(388, 107, 55)	(404,92,54)	(0,0,0)
10	(0, 0, 0)	(0, 0, 0)	(398, 89, 55)	(388,100,59)	(0,1,0)
11	(0, 0, 0)	(0, 0, 0)	(21, 36, 20)	(16,23,18)	(0,1,0)
12	(0, 0, 1)	(0, 0, 0)	(21, 72, 3)	(14,59,1)	(0,1,0)
13	(0, 0, 1)	(0, 0, 0)	(19, 60, 10)	(7,55,4)	(0,1,0)
14	(0, 0, 1)	(0, 0, 0)	(21, 30, 9)	(15,34,3)	(0,2,0)
15	(0, 0, 0)	(0, 0, 0)	(17, 50, 3)	(15,46,2)	(0,2,2)
16	(0, 0, 4)	(0, 0, 1)	(21, 67, 12)	(15,62,4)	(0,1,0)
17	(0, 0, 0)	(0, 0, 0)	(362, 96, 42)	(359,95,44)	(0,1,0)

Extended Data Table 3 | Summary of errors on the consensus sequences on each experiment. Experiment 1, represents our iterative alignment method that identifies the consensus sequences using multiple sequence alignment techniques, majority rules and the balancing property. Experiment 2,3 use Nanopolish software to find the consensus sequences.

Work	Random access	Portability	Seq. technology	Seq. error rate	Error correction/detection	Net density(bits/nt)
**Church**[22]	No	No	HiSeq	0.1-0.3%	None	0.83
**Goldman**[23]	No	No	HiSeq	0.1%	Detection	0.33
**Yazdi**[1]	**Yes**	No	Sanger	0.05%	Correction	1.575
**Grass**[24]	No	No	MiSeq	0.1%	Correction	1.14
**Bornholt**[25]	Yes	No	MiSeq	0.1%	None	0.88
**Erlich**[26]	No	No	MiSeq	0.1%	None	1.55
**This work**	**Yes**	**Yes**	**MinION**	**12-15%**	Correction	**1.72**

Extended Data Table 4 | Comparison of existing DNA-based data storage architectures.

Block	Length	Number of reads	Coverage depth		Error rates %		
			Average	Maximum	Mismatches	Insertions	Deletions
1	1,000	201	176.145	192	12.2	1.6254	7.2202
2	1,000	407	315.521	349	15.9	1.6024	9.0292
3	1,000	490	460.375	482	8.5	2.4417	4.4661
4	1,000	100	81.763	87	8.5	2.2162	4.5559
5	1,000	728	688.663	716	9.3	2.1527	5.1013
6	1,000	136	120.907	129	8.9	2.4052	4.7268
7	1,000	577	542.78	566	8.8	2.7580	4.3375
8	1,000	217	199.018	207	9	2.1290	5.0297
9	1,000	86	56.828	75	9.1	2.3809	4.5893
10	1,000	442	396.742	427	10.1	1.9605	5.8083
11	1,000	114	101.826	110	8.8	2.5494	4.6658
12	1,000	174	162.559	169	10.1	2.4656	5.3833
13	1,060	378	352.35	366	8.9	2.6033	4.4759
14	1,000	222	189.918	203	8.1	2.6159	4.0154
15	1,000	236	222.967	232	9.7	2.5380	4.7343
16	1,000	198	182.99	195	11.1	1.7378	6.5517
17	880	254	240.273	250	9.3	2.3326	5.0274

Extended Data Table 5 | Summary of the alignment using BWA software.
